# Experimental Study of Oriented Strand Board Ignition by Radiant Heat Fluxes

**DOI:** 10.3390/polym13050709

**Published:** 2021-02-26

**Authors:** Ivana Tureková, Iveta Marková, Martina Ivanovičová, Jozef Harangózo

**Affiliations:** 1 Department of Technology and Information Technologies, Faculty of Education, Constantine the Philosopher University in Nitra, Tr. A. Hlinku 1, 949 74 Nitra, Slovakia; ivana.turekova@ukf.sk (I.T.); mabumb@gmail.com (M.I.); jozef.harangozo@ukf.sk (J.H.); 2 Department of Fire Engineering, Faculty of Security Engineering, University of Žilina, Univerzitná 1, 010 26 Žilina, Slovakia

**Keywords:** OSB, heat flux density, ignition time, weight loss

## Abstract

Wood and composite panel materials represent a substantial part of the fuel in many building fires. The ability of materials to ignite when heated at elevated temperatures depends on many factors, such as the thermal properties of materials, the ignition temperature, critical heat flux and the environment. Oriented strand board (OSB) without any surface treatment in thicknesses of 12, 15 and 18 mm were used as experimental samples. The samples were gradually exposed to a heat flux of 43 to 50 kW.m^−2^, with an increase of 1 kW.m^−2^. At heat fluxes of 49 kW.m^−2^ and 50 kW.m^−2^, the ignition times are similar in all OSB thicknesses, in contrast to the ignition times at lower heat fluxes. The influence of the selected factors (thickness and distance from the heat source) was analysed based on the experimentally obtained data of ignition time and weight loss. The experimentally determined value of the heat flux density was 43 kW.m^−2^, which represented the critical heat flux. The results show a statistically significant effect of OSB thickness on ignition time.

## 1. Introduction

Composite panel materials are important wood products [1]. Their production encompasses the utilization of wood of lower quality classes to obtain suitable materials with improved physical and mechanical properties [2]. Oriented strand board (OSB) belong to this group of products but essentially, these products are input materials in the furniture and construction industries [3].

The production of wood-based sheet materials utilizes wood of lower quality classes and chemically safe recyclates and generates materials with improved physical and mechanical properties compared to raw wood [4]. Oriented strand boards (OSBs) are defined in [5] as multilayer boards made of wood strands of a specific shape, thickness and adhesiveness. Strands in the outer layers are oriented parallel to the length or width of the boards. Strands in the middle layer or layers may be oriented randomly or generally perpendicular to the strands of the outer layers [5].

Oriented strand board, also known as OSB, waferboard, Sterling board or Exterior board and SmartPly, is a widely used engineered wood product formed by strands (flakes) of wood, often layered in specific orientations [6]. In appearance, it may have a rough and variegated surface with the individual strands (typically around 2.5 by 15 cm each) lying unevenly across each other. OSBs are cheap and strong boards, and this makes them excellent building material [7].

OSB is produced from thinner wood strands, which can be arranged better with respect to the direction of the wood fibres when adding the layers in longitudinal and transverse directions in the production flow. Strands have the longest length in the direction of the fibres [8]. Thinner debarked forest wood assortments, predominantly soft deciduous woods but also coniferous woods, are used as a raw material for OSB production. Great emphasis is placed on the preparation of strands. They are mainly produced by disc and drum chippers [9]. Thinner and longer strands are used for the surface layer, thicker and slightly shorter strands are used for the middle layers. Fine particles and dust are carefully sorted out. The strands are pressed under high pressure and temperature, using formaldehyde-based synthetic resins [10].

Most commonly used adhesives are conventional synthetic adhesives, both liquid and powder, including isocyanate-based adhesives [11]. OSB is categorized based on the purpose of its application. General-purpose boards (manufactured with a thickness of 9, 11, 15 or 18 mm) and boards for indoor furnishing (including furniture) in dry conditions are classified as OSB/1; load-bearing boards for use in dry conditions are classified as OSB/2; load-bearing boards for use in humid conditions are OSB/3; and heavy-duty load-bearing boards for use in humid conditions are OSB/4 [12].

OSB panel product in terms of physical and health hazards is unclassified according to safety data sheets [13]. OSB is the flammable composite material. OSB can be in contact with heat sources, and it will react to the effect of heat and temperature rise in its structure [14,15]. Wood materials are thermal insulators and do not conduct heat; hence there is a gradual process of thermal degradation, which can result in ignition and fire [16,17]. In the case of thermal stress, the strength of OSB decreases with increasing temperature and time of its action, while at higher temperatures the rate of this change is higher [18].

Flammability is defined as the ability of a sample to ignite under the action of an external thermal initiator and under defined test conditions according to [19]. According to International Organization for Standardization (ISO) 3261 [20], flammability is the ability of a material to ignite. Flammability is characterized by the ignition time of substances and materials, which depends on the ignition temperature, thermal properties of materials, sample conditions (size, humidity, orientation) and critical heat flux [21]. The definition of the term “ignition temperature” can be interpreted as the minimum temperature to which the air must be heated so that the sample placed in the heated air environment ignites, or the surface temperature of the sample just before the ignition point [22,23]. This interpretation was used as the basis of our research. The results were realized on tested equipment without a small burner flame, having only a radiated heat loading.

The aim of this article is to monitor the significant effect of heat flux density (from 43 to 50 kW.m^−2^) and thickness (12 mm, 15 mm, 18 mm) of OSB on the ignition time and change in the weight of the sample. The change in heat flux was previously monitored and recorded for the purpose of validating equipment for the research of heat release loading.

At the same time, the critical temperature of the ignition point of the OSB was experimentally determined depending on the time of action of the radiant heat source and the intensity of the heat flux.

## 2. Materials and Methods

### 2.1. Experimental Samples

**Figure 1:**
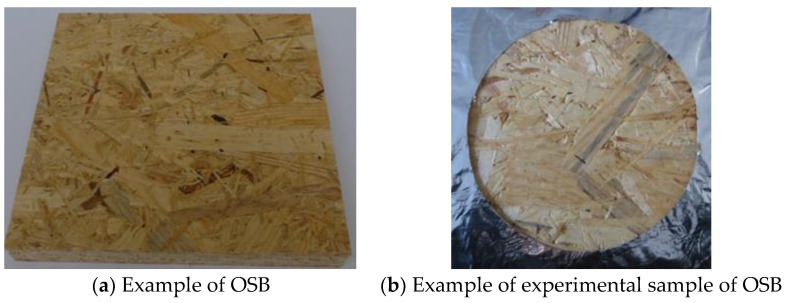
Oriented strand board in (**a**) full piece and (**b**) prepared for measurement according to [24].

Samples of oriented strand board (OSB) without surface treatment, produced by Kronospan Jihlava (KRONOSPAN CR, spol. s r.o., Jihlava, Czech Republic) under the title OSB/3 SUPERFINISH ECO (Figure 1), were selected for the experiments. The OSBs used were multilayer boards made of flat strands of a specific shape and thickness. The strands in the outer layers were oriented parallel to the length or width of the board, and the strands in the middle layers were oriented randomly or were generally perpendicular to the lamellas of the outer layers. They were bonded with melamine formaldehyde resin and polymeric diphenylmethane diisocyanate (PMDI) (Shandong Shanshi Chemical Co Ltd, Zhangdian District, Zibo City, Shandong Province, China) and they were flat-pressed. The boards contained mainly a mixture of coniferous wood. Table 1 shows the physical and chemical properties and fire technical characteristics of the OSB with thickness of 10 mm to 18 mm [10,13].

Samples of OSB were cut to specific dimensions (165 × 165 mm) according to ISO 5657: 1997 [24]. The density of the strand boards was determined according to Standard EN 323: 1996 [25].

The selected board materials were stored at a specific temperature (23 °C ± 2 °C) at relative humidity (50 ± 5%). Rantuch et al. [30] state that OSB moisture does not have a significant effect on the critical conditions under which they can be ignited, but significantly affects the time at which it occurs.

### 2.2. Experimental Procedures

The methodology can be divided into three parts:

1. Verification of test equipment; a small burner flame was not used as the secondary ignition source.
2. Determination of ignition time and weight loss depending on the selected level of heat flux density and thickness of board materials and on the distance of selected board materials from the ignition source.
3. Determination of the critical temperature during ignition of an OSB with a thickness of 15 mm.

#### 2.2.1. Verification of Test Equipment

The beginning of the experimental measurement entailed verification of the test equipment according to ISO 5657: 1997 [24]. The aim of this verification was to monitor the temperature parameters of the cone calorimeter. The cone calorimeter must provide a heat flux in the range of 10 to 70 kW.m^−2^ in the centre of the hole in top plate and in a base plate which matches with the bottom side of the top plate (Figure 1b).

Verification of the actual condition of the cone calorimeter was performed using a SBG01-100 radiometer with a cooling sensor (Hukseflux, Delft, The Netherlands) and a METEX M-3890D digital multimeter (Manufacturer: Zebronics, India) with a USBVIEV program (SOFTONIC INTERNATIONAL S.A., Barcelona, Spain). Several experimental measurements, which included collecting voltage values from the multimeter and evaluating the results using the calibration curve specified in the standard, confirmed that at the selected temperature, the cone calorimeter indicated the corresponding heat flux densities in accordance with the previous measurements performed during test equipment calibration.

**Figure 2:**
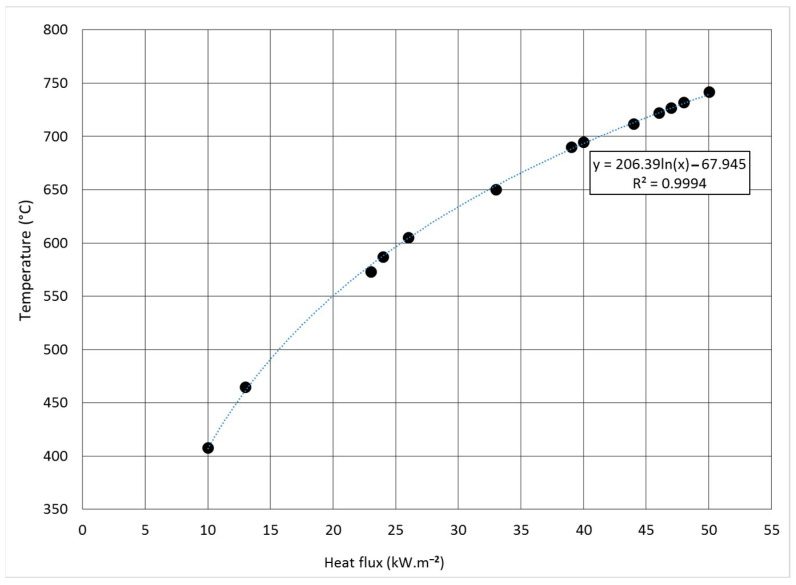
Heat flux dependence on cone calorimeter temperature.

Based on the obtained values and compared results, a graphical dependence of the heat flux on the temperature of the cone calorimeter was constructed (Figure 2). The measurements were repeated with each thickness of the board material 5 times.

#### 2.2.2. Methodology for Determining Ignition Time and Weight Loss

**Figure 3:**
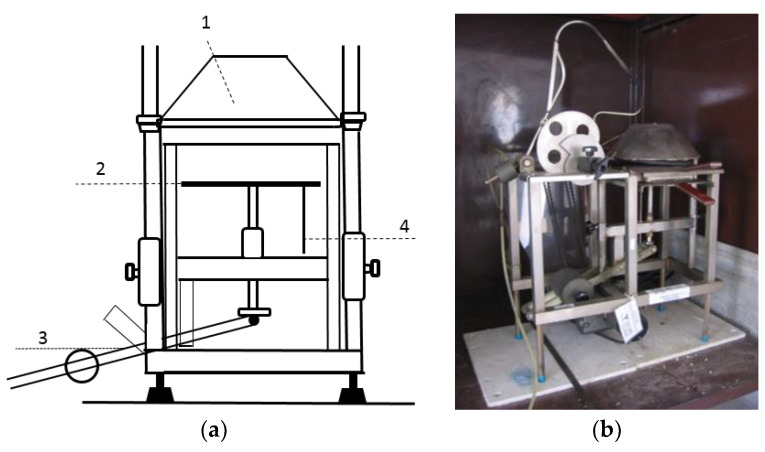
(**a**) Scheme of the equipment for determination of flammability of materials at a heat flux of radiant heat of 10–50 kW.m^−2^ according to [24]; (**b**) a photo of the real test equipment [31]. Legend: 1, heating cone; 2, board for sample; 3, movable arm; 4, connection point for recording experimental data.

The ignition time and weight loss depending on the selected level of heat flux density and thickness of the board materials, as well as on the distance of the selected board materials from the ignition source, were determined according to a modified procedure based on ISO 5657: 1997 [24]. This modification included a change of igniter. Ignition was caused only by heat flux, without the use of a direct flame (Figure 3a).

The samples were placed horizontally and exposed to a heat flux of 43 to 50 kW.m^−2^ by an electrically heated conical radiator (Beijing Global Trade Software Technology Co., Ltd., Chaoyang District, Beijing, China). Orientation experiments determined the minimum heat flux required to maintain flame combustion. The time-to-ignition value was recorded, while considering only the permanent ignition of the surface of the analysed sample when exposed to a selected level of heat flux density.

The observed factors influencing the ignition time and weight loss were

- the thickness of the board material; and
- the density of the radiant heat flux.

#### 2.2.3. Determination of the Critical Ignition Temperature

Determination of the critical ignition temperature was performed on a sample of OSB with a thickness of 15 mm. Test equipment according to [24] was supplemented by two thermocouples. These thermocouples were placed in the middle of the top and bottom surfaces of the board.

Test samples were exposed to four selected levels of heat flux density (44, 46, 48 and 50 kW.m^−2^) for 300 s, which was repeated 5 times.

#### 2.2.4. Statistical Processing of Data and Evaluation of Results

To evaluate the influence of the above-mentioned factors on the ignition temperature and weight loss, the obtained results were subjected to statistical analysis. The obtained results of the ignition and weight loss temperatures were statistically evaluated by one-way analysis of variance (ANOVA) using the Least Significant Difference LSD test (95%, 99% detectability level) (STATGRAPHICS software version 18/19, The Plains, VA, United States), with the use of board material thickness (12, 15 and 18 mm) and radiant heat flux density (from 43 to 50 kW.m^−2^) as influence factors.

## 3. Results and Discussion

**Figure 4:**
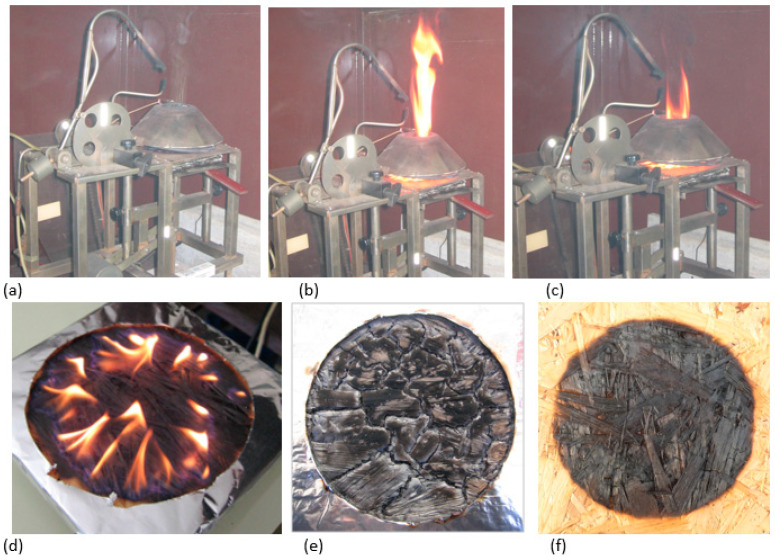
Demonstration of the course of burning of oriented strand boards (OSBs) after their ignition by radiant heat (**a**) ignition (100 s); (**b**) burning time of 160 s; and (**c**) 300 s. (**d**) A demonstration of the combustion process by 44 kW.m^−2^. (**e**) A sample selected from the measuring device; specific distance of 12 mm, time of ignition of 57 s and density heat flux of 50 kW.m^−2^. (**f**) Cooled sample 10 min after the experiment; sample thickness of 15 mm and ignition time of 108 s, 44 kW.m^−2^.

The course of the experiment (Figure 4) according to [24] confirmed the verified behaviour of the material in terms of the classification “reaction to fire D-s1, d0 by [10]”. D-s1, d0 means “combustible materials – medium contribution to fire, with speed of emission absent or weak during combustion” by [26]. The priority of the experiment was to monitor the critical parameters of the ignition based on the change in board thickness (Table 2).

### 3.1. Determination of Ignition Temperature and Weight Loss

For the purposes of our research, the initial value of the radiant heat flux, to which the selected OSBs were exposed, was experimentally set to 43 kW.m^−2^. This value represented the critical heat flux for the selected samples. Critical heat flux is the heat flux between the minimum incident heat fluxes at which ignition occurs and maximum incident heat flux at which ignition does not occur. It can be used to evaluate the ignition ability. The critical heat flux is obtained experimentally by gradually exposing the samples to decreasing heat flux until ignition ceases [30]. The maximum value of the radiant heat flux, to which selected board materials were exposed, was 50 kW.m^−2^. The heat flux gradually increased by 1 kW.m^−2^ (Table 2).

The effect of external heat flux on a sample is the incident energy upon its surface. Part of the energy is reflected (depending on the emissivity of the surface), part is transferred by conduction to deeper layers of the material (depending on its thermal conductivity) and the rest is absorbed by a thin layer on the surface, i.e., heating takes place. As the temperature of the sample increases, pyrolysis and thermal oxidation occur, as a result of which gaseous products are released into the environment [30]. Therefore, the time to ignition of the sample decreases with increasing external heat flux [31], which is confirmed by our results.

An interesting result is the reduction of time differences in individual thicknesses at higher heat fluxes. Heat fluxes of 49 and 50 kW.m^−2^ offered almost equal times in all thicknesses. Differences in ignition times decreased with increasing heat flux, and the ignition differences between the individual thicknesses decreased. The maximum difference of ignition time between thicknesses (67.4 s) was at 43 kW.m^−2^ and the minimum (3.4 s) was at 50 kW.m^−2^.

It can therefore be concluded that with increasing heat flux value, the thickness of the OSB has no significant effect on the ignition time. The increase in weight loss was with increasing heat flux, namely in 12 mm by 5.3%, in 15 mm by 2.9% and in 18 mm by 2.41%.

With increasing heat flux, there was an increase in weight loss of 12 mm by 5.3%, 15 mm by 2.9% and 18 mm by 2.41%. It is possible to make the following assumption: smaller thicknesses of OSB due to the increase of heat flow have more intensive thermodegradation processes, which manifest in higher weight losses.

Mitterová and Garaj [32] studied the weight loss and ignition time of an OSB caused by the action of radiant heat. Test samples were exposed to an infrared heater with a power of 1000 W for 600 s, while the distance of the samples from the surface of the radiating body was 30 mm.

The weight loss of the OSB reached 58.92%, and the ignition time was 53.4 s. In comparison with the values obtained by our research, the results are significantly different, because the distance between the emitters and the sample was relatively high, which affected the ignition time. The surprising result is more than 50% weight loss under these conditions.

**Figure 5:**
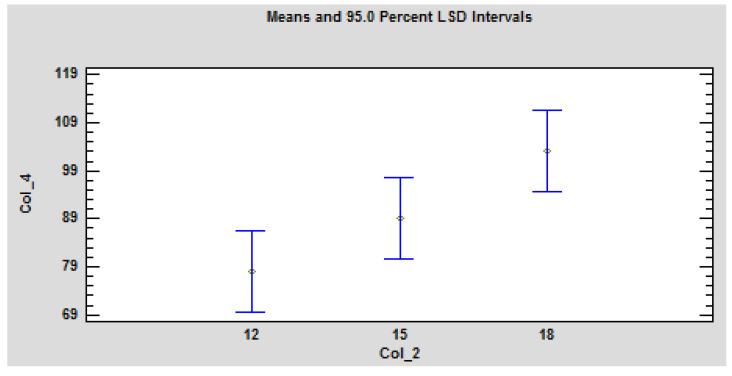
Graphical representation of the statistical evaluation of the influence of the board thickness on the ignition time under the action of the radiant heat source on the OSB. Legend: Col_2, thickness; Col_4, Time-to-ignition.

The use of one-way ANOVA confirms the significant dependence of the ignition time on the board thickness (Table 3, Figure 5). The ANOVA table decomposes the variance of Col_4: Time- to-ignition into two components: a between-group component and a within-group component. The F-ratio, which in this case equals 4.31448, is a ratio of the between-group estimate to the within-group estimate. Since the P-value of the F-test is less than 0.05, there is a statistically significant difference between the mean Col_4 from one level of Col_2 to another at the 5% significance level. To determine which means are significantly different from which others, select Multiple Range (Table 3).

**Figure 6:**
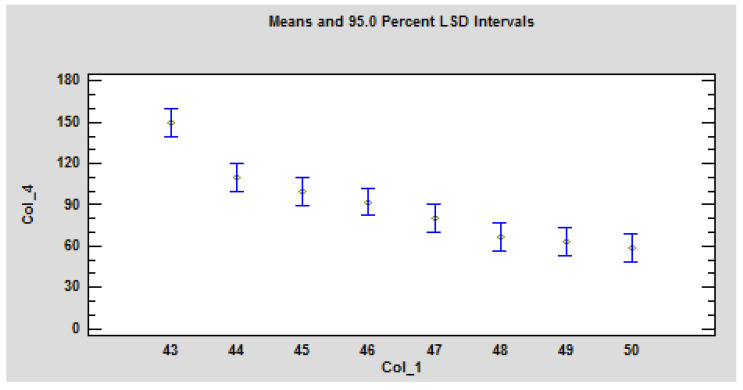
Graphical representation of the statistical evaluation of the influence of heat flux on ignition time under the action of a radiant heat source on the OSB. Legend: Col_1, heat flux; Col_4, Time-to-ignition.

Concurrently, a significant dependence of the ignition time on the heat flux was confirmed (Table 4, Figure 6). The ANOVA table decomposes the variance of Col_4 into two components: Time-to-ignition and heat flux. The F-ratio, which in this case equals 17.5456, is a ratio of the between-group estimate to the within-group estimate. Since the P-value of the F-test is less than 0.05, there is a statistically significant difference between the means Col_4 Time-to-ignition from one level of Col_1 heat flux to another at the 5% significance level. To determine which means are significantly different from which others, select Multiple Range (Table 4).

**Figure 7:**
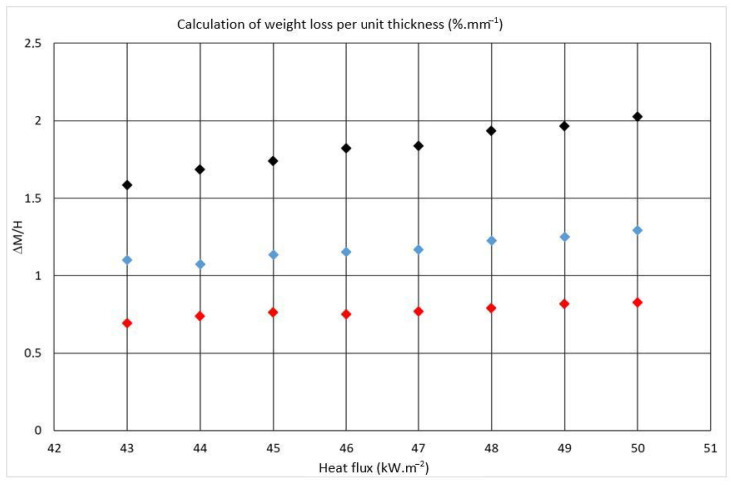
Graphical dependence of the weight loss ΔM and sample thickness H ratio on the heat flux. Legend: black point, 12 mm thickness; blue point, 15 mm thickness; red point, 18 mm thickness.

Weight loss caused by thermal degradation of the board surface, which was exposed to radiant heat (Table 2), maintained the same course. Trend lines for the individual board thicknesses have a parallel direction, so it can be assumed that the weight loss is uniform per each unit of thickness (Figure 7). Difference can be seen in the samples with a thickness of 12 mm.

The results clearly show the following dependence: with increasing thickness, the weight loss decreases, but the ratio of weight loss and sample thickness ΔM/H is rather similar in all heat fluxes (Figure 7). A similar scenario is observed at a thickness of 15 mm. The highest weight loss is observed at a thickness of 12 mm, where an area of light weight of the material burns down and it is possible to observe a slight increase in weight loss with increasing heat flux. There are several studies that confirm the reduction of the weight loss and prolongation of time-to-ignition by appropriate fire-retardant treatment of the boards [3,33,34].

Statistical analysis confirmed this fact. The heat flux (Table 5) and material thickness (Table 6) have no statistically significant effect on the change in sample weight.

The ANOVA table decomposes the variance of Col_3 Weight loss (%) into two components: a between-group component and a within-group component. The F-ratio, which in this case equals 2.0454, is a ratio of the between-group estimate to the within-group estimate. Since the P-value of the F-test is greater than or equal to 0.05, there is not a statistically significant difference between the mean Col_3 Mass loss (%) from one level of Col_1 (heat flux) to another at the 5% significance level.

Additionally, since the P-value of the F-test is greater than or equal to 0.05, there is not a statistically significant difference between the mean Col_3 Weight loss (%) from one level of Col_2 thickness to another at the 5% significance level.

### 3.2. Determination of Critical Ignition Temperature

Critical ignition temperature was monitored by means of thermocouples placed below and above the test sample. The increase in temperature was monitored on the upper surface of the 15 mm OSB, which was exposed to a direct radiant heat flux of 44, 46, 48 and 50 kW.m^−2^ for 300 s. The OSB boards with 15 mm thickness are the most commonly used building material in the construction of soffits.

**Figure 8:**
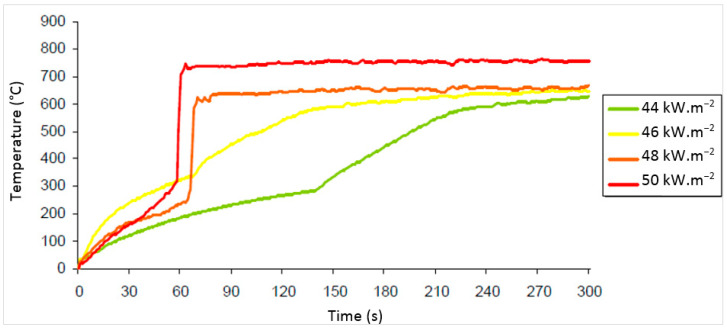
Temperature dependence of the upper surface of an OSB with a thickness of 15 mm on the exposure time to the cone calorimeter.

The first set of experiments with a heat flux density of 44 kW.m^−2^ for the period of 300 s resulted in an ignition time of 142 s (Table 7). The temperature at the bottom surface of the board rose minimally (Figure 8). The time to ignition (142 s) was the longest compared to other values obtained at higher heat fluxes (Table 3). The ignition temperatures obtained from the experiments varied. It is not possible to find the correlation with other parameters.

The temperature at the upper surface of the sample rose sharply to about 700 °C after ignition (Figure 8). The temperature at the bottom surface of the sample rose slowly and had a comparable course in all heat fluxes (Figure 8).

The temperature curves have a smooth linear course and show dependence of the temperature at the upper surface on the time of exposure to radiant heat with heat fluxes of 44 kW.m^−2^ and 46 kW.m^−2^ (Figure 8). After ignition, there was a sharp rise in temperature at the upper surface of the sample (Figure 8). The sharp rise in temperature was likely caused by two effects. The first effect was a higher heat flux from the cone calorimeter to the surface of the tested sample and the second effect was a higher reverse heat radiation from flares [35].

Rantuch et al. [21] divided the OSB combustion process into five phases based on extensive research on OSB (14 mm thickness) using a cone calorimeter with heat fluxes of 20, 30, 40, 50 and 60 kW.m^−2^. The first phase entails a period prior to the sample ignition. The heat received by the samples is consumed while heating them, but the concentration of gaseous combustible products during thermal degradation is not sufficient to initiate the flame combustion. The second phase occurs after ignition of the sample. The amount of released flammable gases is sufficient to maintain a steady-flame combustion and at the same time a carbonized layer has not yet formed on the surface of the OSB. The mass burning rate as well as the rate of heat release have, therefore, high values. After the formation of a carbonized layer (Figure 4e) on the exposed surface of the samples, a steady burning phase occurs. The rate of heat release is almost constant, and the weight loss is uniform. This is followed by the burning phase of the superheated sample, characterized by significant pyrolysis in its entire volume (Figure 4d).

The increase in the concentration of pyrolysis products is manifested by a more pronounced combustion and, thus, also by an increase in the rate of heat release. After complete burnout of the gaseous pyrolysis products, flameless combustion of the sample occurs with low weight loss [21].

OSB is often a part of the structural elements in the exterior, and therefore it is also crucial to carry out large-scale fire tests [36,37,38]. Sultan [36] showed that the effect of insulation types on the fire resistance of exterior wall assemblies with OSB sheathing can be considered significant for specific conditions.

## 4. Conclusions

The ignition process cannot be defined by a single characteristic. Our research monitored important parameters, specifically type of the sample (OSB), thickness, heat flux density, weight loss, ignition temperature and critical ignition temperature, and modified conditions specified in the test procedures. OSBs with a thickness of 12, 15 and 18 mm were analysed with a focus on the critical heat flux and the ignition temperature. The experimentally determined value of the heat flux density was 43 kW.m^−2^, which represented the critical heat flux. In all cases, dependences of the ignition time on the external heat flux were confirmed and there was a correlation between the ignition time, weight loss and intensity of radiant heat.

The following results were obtained from the conducted experiments:

- As the heat flux density increased, the ignition time decreased in all thicknesses of analysed OSB.
- The ignition time increased with increasing thickness of the OSB, and the weight loss decreased with increasing thickness of the OSB at a constant heat flux.
- With increasing board thickness, the weight loss decreased. The largest average weight loss of 24.31% was recorded in a 12 mm OSB that was exposed to a radiant heat flux of 50 kW.m^−2^; the lowest average weight loss of 12.44% was recorded in a sample of 18 mm OSB that was exposed to a radiant heat flux of 43 kW.m^−2^.
- The ignition time is significantly dependent on the thickness of the OSB sample and on the value of the heat flux. As the heat flux increases, the ignition time shortens; as the thickness of the OSB increases, the ignition time extends. The largest weight loss of 27.22% was recorded in an OSB with a thickness of 12 mm and a density in the range of 500 to 550 kg.m^−3^, which was exposed to a radiant heat flux of 50 kW.m^−2^. The lowest weight loss of 11.91% was found in an OSB with a thickness of 18 mm and a density in the range of 550–600 kg.m^−3^, which was exposed to a radiant heat flux of 43 kW.m^−2^.
- The weight loss is not significantly dependent on the ignition time and the thickness of the OSB.
- Critical temperature of an OSB with a thickness of 15 mm that was exposed to heat flux densities of 44 kW.m^−2^ and 46 kW.m^−2^ had a linear character; at the heat flux densities of 48 kW.m^−2^ and 50 kW.m^−2^, it had an initially linear course, but due to ignition, a sharp rise in temperature was noted at the upper surface of the sample. The sharp rise in temperature was caused by two effects. The first effect was a higher heat flux from the cone calorimeter to the surface of the tested sample, and the second effect was a higher reverse heat radiation from flares.

Analysed parameters, such as the time-to-ignition parameter, related weight loss of the OSBs, the density and thickness of the OSBs, radiant heat flux density, distance of the ignition source from the material and determination of the critical ignition temperature with a modified arrangement of the test equipment and horizontal placement of the sample, confirmed the importance and complexity of these parameters for a better understanding of the critical ignition conditions of the OSB, as well as the combustion process.

## Figures and Tables

**Table 1:** Physical and chemical properties and fire technical characteristics of the OSB with thickness of 10–18 mm [13].

Parameters	Notes	Values
Density (kg.m^−3^)		630 ± 10%
Humidity (%)		5 ± 12%
Bending Strength (N.mm^−2^)	Main Axis	20
	Secondary Axis	10
Modulus of Elasticity (N.mm^−2^)	Main Axis	3500
	Secondary Axis	1400
Swelling (%)		15
Thermal Conductivity (W.m^−2^.K^−1^)		0.13
Formaldehyde Content (mg.100 g^−1^)		8
Flame Spread Index ^1^		83.3
Reaction to Fire	Thickness 9 mm ^2^	D-s2, d2
	Thickness 18 mm ^3^	D-s1, d0
Class of Fire Reaction [26]		E—Eoderately Flammable

^1^ Flame spread index was calculated from peak heat release rate (HRR), total heat release and time to sustained ignition by ASTM E 84 ([27] and [28]; ^2^ [29]; ^3^ [10].

**Table 2:** Ignition time and weight loss in samples with different thicknesses using heat fluxes of 40 to 50 kW.m^−2^ at a distance of 20 mm.

Density of Radiant Heat Flux (kW.m^−2^)	Corresponding Temperature (°C) ^1^	Thickness (mm)	Ignition Time (s)	Weight Loss (%)
43	700	12	107.4 ± 32.927	19.018 ± 0.742
		15	172.8 ± 68.271	16.528 ± 1.103
		18	170.0 ± 19.279	12.436 ± 0.402
44	710	12	80.80 ± 14.372	20.188 ± 1.210
		15	108.0 ± 31.093	16.092 ± 0.885
		18	140.0 ± 31.698	13.256 ± 0.745
45	720	12	86.4 ± 10.442	20.87 ± 0.889
		15	100.2 ± 21.673	17.026 ± 0.541
		18	111.2 ± 24.235	13.716 ± 0.303
46	724	12	84.4 ± 9.002	21.868 ± 0.879
		15	93.4 ± 21.767	17.272 ± 0.647
		18	98.8 ± 12.592	13.504 ± 0.228
47	727	12	67.08 ± 5.403	22.026 ± 0.908
		15	71.0 ± 8.671	17.5 ± 0.455
		18	103.6 ± 18.391	13.818 ± 0.266
48	730	12	58.60 ± 5.953	23.206 ± 0.505
		15	63.40 ± 7.116	18.366 ± 0.910
		18	77.60 ± 25.881	14.222 ± 0.826
49	735	12	62.20 ± 3.2497	23.578 ± 0.858
		15	63.20 ± 3.187	18.764 ± 0.571
		18	65.0 ± 11.436	14.678 ± 0.899
50	742	12	56.80 ± 2.039	24.302 ± 0.814
		15	59.40 ± 5.607	19.402 ± 0.586
		18	60.20 ± 5.741	14.846 ± 1.033

^1^ Based on the graphical dependence in Figure 3.

**Table 3:** ANOVA table for Col_4 Time-to-ignition by Col_2 thickness.

Source	Sum of Squares	Df	Mean Square	F-Ratio	P-Value
Between Groups	12,608.1	2	6304.03	4.31	0.0156
Within Groups	170,953	117	1461.13		
Total (Corr.)	183,561	119			

**Table 4:** ANOVA table for Col_4 Time-to-ignition by Col_1 heat flux.

Source	Sum of Squares	Df	Mean Square	F-Ratio	P-Value
Between Groups	96,009.1	7	13,715.6	17.55	0.000
Within Groups	87,551.5	112	781.71		
Total (Corr.)	183,561	119			

**Table 5:** ANOVA table for Col_3 Weight loss by Col_1 heat flux.

Source	Sum of Squares	Df	Mean Square	F-Ratio	P-Value
Between Groups	5.11703E7	7	7.31005E6	2.05	0.0554
Within Groups	4.00277E8	112	3.5739E6		
Total (Corr.)	4.51448E8	119			

**Table 6:** ANOVA table for Col_3 Weight loss by Col_2 thickness.

Source	Sum of Squares	Df	Mean Square	F-Ratio	P-Value
Between Groups	5.78793E6	2	2.89396E6	0.76	0.4701
Within Groups	4.4566E8	117	3.80906E6		
Total (Corr.)	4.51448E8	119			

**Table 7:** Time-to-ignition and ignition temperature at the upper and lower surfaces of a 15 mm thick OSB sample corresponding to the individual radiant heat flux densities.

Heat Flux (kW.m^−2^)	Time to Ignition (°C)	Temperature (°C)	
		Direct Side of Heat	Opposite Side
44	142	287	34
46	70	358	25
48	64	252	26
50	58	319	27

## Data Availability

Not applicable for studies not involving humans or animals.
